# The primary transcriptome of the fast-growing cyanobacterium *Synechococcus elongatus* UTEX 2973

**DOI:** 10.1186/s13068-018-1215-8

**Published:** 2018-08-04

**Authors:** Xiaoming Tan, Shengwei Hou, Kuo Song, Jens Georg, Stephan Klähn, Xuefeng Lu, Wolfgang R. Hess

**Affiliations:** 1 Key Laboratory of Biofuels, Shandong Provincial Key Laboratory of Synthetic Biology, Qingdao Institute of Bioenergy and Bioprocess Technology, Chinese Academy of Sciences, No. 189 Songling Road, Qingdao, 266101 China; 2 Genetics and Experimental Bioinformatics, Faculty of Biology, University of Freiburg, Schänzlestraße 1, 79104 Freiburg, Germany; 3 Department of Solar Materials, Helmholtz Centre for Environmental Research-UFZ, Permoserstraße 15, 04318 Leipzig, Germany; 4 Laboratory for Marine Biology and Biotechnology, Qingdao National Laboratory for Marine Science and Technology, No. 1 Wenhai Road, Qingdao, 266237 China; 5 Freiburg Institute for Advanced Studies, University of Freiburg, Albertstraße 19, 79104 Freiburg, Germany; 6 Present Address: College of Life Sciences, Hubei University, 368 Youyi Avenue, Wuchang District, Wuhan, 430062 China

**Keywords:** Primary transcriptome, dRNA-Seq, Cyanobacterium, *Synechococcus elongatus* UTEX 2973, Stress tolerance

## Abstract

**Background:**

Cyanobacteria have shown promising potential for the production of various biofuels and chemical feedstocks. *Synechococcus elongatus* UTEX 2973 is a fast-growing strain with pronounced tolerance to high temperatures and illumination. Hence, this strain appears to be ideal for the development of photosynthetic biotechnology. However, molecular insights on how this strain can rapidly accumulate biomass and carbohydrates under high-light and high-temperature conditions are lacking.

**Results:**

Differential RNA-Sequencing (dRNA-Seq) enabled the genome-wide identification of 4808 transcription start sites (TSSs) in *S. elongatus* UTEX 2973 using a background reduction algorithm. High light promoted the transcription of genes associated with central metabolic pathways, whereas the highly induced small RNA (sRNA) PsrR1 likely contributed to the repression of phycobilisome genes and the accelerated glycogen accumulation rates measured under this condition. Darkness caused transcriptome remodeling with a decline in the expression of genes for carbon fixation and other major metabolic pathways and an increase in the expression of genes for glycogen catabolism and Calvin cycle inhibitor CP12. Two of the identified TSSs drive the transcription of highly abundant sRNAs in darkness. One of them is widely conserved throughout the cyanobacterial phylum. Its gene is fused to a protein-coding gene in some species, illustrating the evolutionary origin of sRNAs from an mRNA 3′-end.

**Conclusions:**

Our comprehensive set of genome-wide mapped TSSs, sRNAs and promoter activities will be valuable for projects requiring precise information about the control of transcription aimed at metabolic engineering and the elucidation of stress acclimation mechanisms in this promising strain.

**Electronic supplementary material:**

The online version of this article (10.1186/s13068-018-1215-8) contains supplementary material, which is available to authorized users.

## Background

In recent years, great efforts have been made towards the sustainable production of biofuels, sugars and chemical feedstocks by cyanobacteria. Selected model strains, such as *Synechocystis* sp. PCC 6803 and *Synechococcus elongatus* (hereafter *S. elongatus*) PCC 7942, have been metabolically engineered to produce dozens of different metabolites based on their ability to fix carbon dioxide using light energy [1–3]. Hence, cyanobacteria have shown promising potential not only for the production of these compounds but also for carbon footprint reduction, which is relevant in the context of global warming. However, when compared to microorganisms such as *E. coli* and yeast, which were established earlier for industrial applications, cyanobacterial hosts are commonly limited by their relatively slow growth rates.

*Synechococcus elongatus* UTEX 2973 is a recently characterized cyanobacterial strain with the fastest measured growth rate until now and good tolerance to high temperature and illumination [4]. This strain showed its fastest growth rate at 41 °C under continuous illumination of 500 μmol photons/m^2^/s, which is lethal to other popular model cyanobacterial strains, including *Synechocystis* sp. PCC 6803, *S. elongatus* PCC 7942 and *Synechococcus* sp. PCC 7002 [4]. In addition, *S. elongatus* UTEX 2973 can accumulate glycogen to more than 50% of dry cell mass when nitrogen was replete, whereas a similar glycogen content was reported for other cyanobacteria only under nitrogen-limited conditions [5]. After the introduction of a heterologous sucrose transporter, a mutant of *S. elongatus* UTEX 2973 effectively secreted sucrose at a rate of 35.5 mg/L/h under salt stress conditions, proving that *S. elongatus* UTEX 2973 is a promising candidate to serve as a photosynthetic cell factory [5]. Recently, CRISPR-Cas9 mediated genomic engineering has been established in this strain, which will make metabolic engineering of *S. elongatus* UTEX 2973 more efficient in future [6].

The genomic sequence of *S. elongatus* UTEX 2973 shows only a few differences from that of *S. elongatus* PCC 7942. These differences consist of 55 single-nucleotide polymorphisms, a 188.6 kb inversion and a deletion of 6 open reading frames [4]. These minor genetic differences were also confirmed at the amino acid level by shotgun proteomic analysis [4]. Differences in carbon uptake rates were identified as a major factor in the divergent growth rates between *S. elongatus* UTEX 2973 and PCC 7942 [7] based on flux balance analysis. Despite several existing genomic, proteomic and metabolomics studies on *S. elongatus* UTEX 2973, it has remained enigmatic how this special strain achieves fast photosynthetic carbon fixation under high-light and high-temperature conditions. Gene expression modulation may be an important factor in this special physiological capability.

Microarray and RNA-Seq analyses have been extensively applied to evaluate the effects of abiotic stresses on the transcriptomic profiles of cyanobacteria [8–11]. These studies have mainly focused on the model organisms *Synechocystis* sp. PCC 6803 and *S. elongatus* PCC 7942. Unlike microarray and classical RNA-Seq, differential RNA-Seq (dRNA-Seq) allows for the precise identification of active transcription start sites (TSSs) for both mRNAs and non-coding RNAs (small regulatory RNAs, sRNAs) on top of providing information on mRNA levels [12]. Hundreds of sRNA candidates were identified in 7 different cyanobacterial strains with dRNA-Seq analysis [13]. A large number of unknown regulators, including several sRNAs, were identified by comparing the primary transcriptome of *Anabaena* sp. PCC 7120 with and without nitrogen starvation [14]. The same approach has been prolific in the analysis of the response of *Synechocystis* sp. PCC 6803 [15] and PCC 6714 [16] to 10 different environmental stimuli. Several antisense RNAs (asRNAs) and sRNAs with important physiological functions and special regulatory mechanisms were identified from the precise TSS information provided by dRNA-Seq [17–20].

In this study, we performed genome-wide TSS prediction at single-nucleotide resolution for *S. elongatus* UTEX 2973 and determined 4808 TSSs using RNA from cells grown under standard, high light, high light coupled with high-temperature and dark conditions. We identified the major regulatory mechanisms of efficient biomass production under high-light conditions by comparing the differential accumulation of primary reads driven by specific promoters. Moreover, we identified two sRNAs that are highly abundant in darkness, which suggests that they might have a function linked to light–dark transitions. Our comprehensive information will favor the further research on the stress acclimation mechanisms of cyanobacteria, and provide molecular basis for the metabolic engineering of *S. elongatus* UTEX 2973.

## Results

### The primary transcriptome of *Synechococcus elongatus* UTEX 2973

#### Transcriptional start sites

**Fig. 1:**
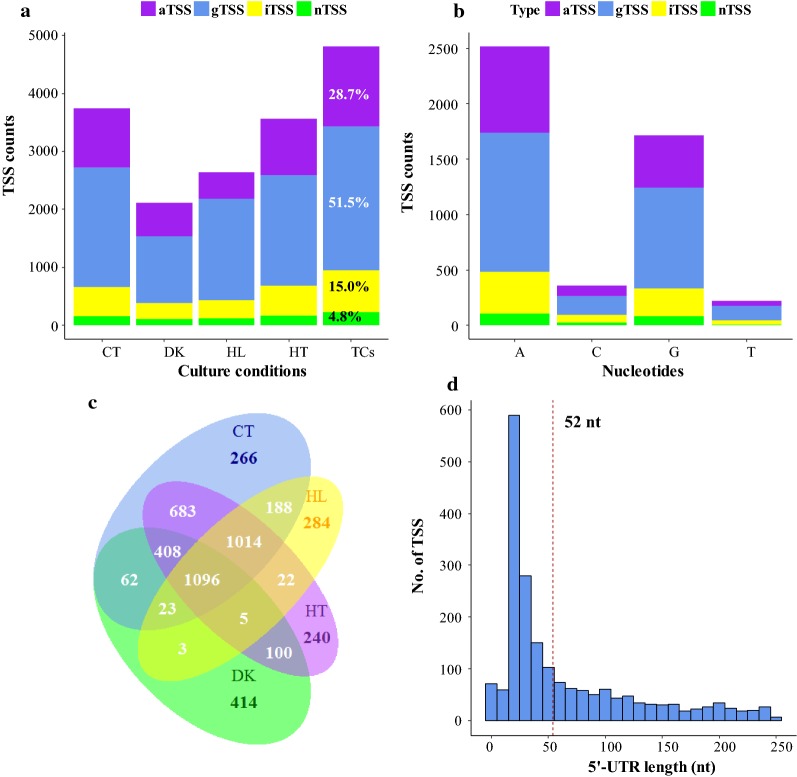
Statistical analysis of the TSSs identified in the transcriptomes of *S. elongatus* UTEX 2973. TSSs with ≥ 300 raw reads were considered to be active and were included in the following statistical analysis. TSSs were classified into four different types according to previous studies [21, 22]: gTSS (blue), aTSS (purple), iTSS (yellow), and nTSS (green). **a** Numbers of each type of TSS identified from the different growth conditions. CT, DK, HL and HT represent control, dark, high-light and high-temperature conditions, respectively. TCs represent the total of all TSSs identified across the four conditions. **b** Nucleotide usages for each TSS type. **c** Distribution of TSSs active under different respective growth conditions. **d** Histogram showing the distribution of 5′-UTR lengths of gTSSs. The median 5′-UTR length is indicated as a dashed line

Total RNA was isolated from cells transferred to darkness or high light for 2 h, maintained at an elevated temperature for 30 min, or maintained in control conditions to identify active TSSs governing the transcriptome of *S*. *elongatus* UTEX 2973 under different conditions. The samples were used to construct and sequence primary libraries that provided information on TSSs, as well as reference libraries, which provide classical and reference transcriptome information, respectively (see “Methods” section for details on the prepared libraries). TSSs were identified genome wide and categorized into gTSSs (TSSs of annotated genes), iTSSs (TSSs of gene-internally starting transcripts), aTSSs (TSSs of antisense RNAs) and nTSSs (TSSs of transcripts from intergenic regions) according to the concepts developed in previous studies [21–23]. The numbers and types of active TSSs under different culture conditions are summarized in Fig. 1a, whereas the full list of all TSSs, including their positions, nucleotides, types and raw read numbers, is listed in Additional file 1: Table S1. In total, 4808 TSSs, including 2475 gTSSs, 1380 aTSSs, 724 iTSSs and 229 nTSSs, were found in at least one of the tested culture conditions (Fig. 1a), yielding on average one TSS per 571 bp. Hence, 51.5% of all TSSs (gTSSs) led to mRNA, which could be translated to a protein, whereas 4.8% (nTSSs) gave rise to sRNAs. *S. elongatus* UTEX 2973 has the highest ratio between the numbers of gTSSs and nTSSs to date compared with seven previously reported cyanobacterial primary transcriptomes with gTSS percentages below 38.0% and nTSSs of more than 5.1% [13].

The initiation nucleotides of each transcript are listed in Additional file 1: Table S1. Transcription in *S. elongatus* UTEX 2973 was preferentially initiated at adenosine residues (52.3%), whereas 35.6% of transcripts began at a guanosine (Fig. 1b). The strong preference for a purine at the first transcribed nucleotide is consistent with previous findings in other cyanobacteria [21, 24].

Only 1096 (22.8%) of the total of 4808 TSSs were found to be active under all four conditions. When darkness was excluded, 2110 TSSs (43.9%) were active under control, high-light and high-temperature conditions. Several TSSs were only identified under a certain condition, i.e., 266, 414, 284 and 240 TSSs were specific to control, dark, high-light and high-temperature conditions, respectively. These TSSs account for 7.1, 19.6, 10.8 and 6.7% of the total number of TSSs identified in control, dark, high-light and high-temperature conditions, respectively. The relatively high share of dark-specific TSSs indicates that a specific set of genes was up-regulated in this condition. The TSSs associated with the highest read counts are listed in Table 1. Among them, 11 TSSs were found to be maximally expressed under darkness (Table 1).

**Table 1:** List of top 20 abundant transcripts in *S. elongatus* UTEX 2973

Gene/sRNA	Type	Strand	Position	Product	Log_2_-Fold change			Max condition^*a*^
					DK/CT	HL/CT	HT/CT	
M744_RS11628	gTSS	−	2,349,022	Hypothetical protein	7.30	− 2.48	3.80	DK
Sye_sRNA3	nTSS	+	1,471,309	ncRNA	4.62	− 5.21	1.27	DK
*hspA*	gTSS	+	788,507	Molecular chaperone	6.91	− 0.30	− 0.23	DK
*ssrA*	nTSS	+	2,274,728	tmRNA	4.03	− 0.21	− 0.06	DK
*psaA*	gTSS	+	1,138,200	Photosystem I P700 chlorophyll a apoprotein A1	− 0.62	− 2.23	0.09	HT
Sye_sRNA1	nTSS	+	1,559,116	sRNA	1.35	− 1.42	− 0.08	DK
*gifA*	gTSS	+	2,354,845	Glutamine synthetase inactivating factor IF7	6.41	− 1.82	− 0.68	DK
*psbI*	gTSS	−	1,491,583	Photosystem II reaction center protein I	− 1.56	0.58	0.68	HT
*rpl10*	gTSS	+	2,633,453	50S ribosomal protein L10	− 1.86	3.67	− 0.67	HL
*rps21*	gTSS	−	1,425,136	30S ribosomal protein S21	− 2.55	2.71	− 0.32	HL
*psbE*	gTSS	+	2,054,473	Cytochrome b559 subunit alpha	− 0.21	− 0.03	− 0.09	HT
*sphX*	gTSS	+	741,520	Protein SphX	− 4.07	2.01	− 1.15	HL
*cp12*	gTSS	+	319,690	Hypothetical protein	6.90	− 0.00	− 0.17	DK
*rbp1*	gTSS	−	2,585,082	RNA-binding protein	4.24	3.26	− 0.14	DK
M744_RS12900	gTSS	+	2,605,244	Hypothetical protein	4.47	− 2.25	0.59	DK
*gltB*	gTSS	−	2,367,947	Glutamate synthase subunit alpha (ferredoxin-GOGAT, Fd-GOGAT)	0.80	0.19	− 0.63	DK
M744_RS00180	gTSS	−	34,832	Hypothetical protein	− 2.95	− 3.64	− 0.25	CT
M744_RS05545	gTSS	+	1,062,162	Ribonuclease III	2.53	2.63	− 0.15	DK
*somA*	gTSS	+	1,561,830	Porin	0.05	2.63	− 0.52	HL
PsrR1	nTSS	+	11,213	sRNA	− 1.98	5.79	− 3.92	HL

All transcripts (except for rRNA) were sorted according to their normalized read counts

^a^The culture conditions under which the level of each transcript reached its maximum

#### 5′-UTRs of coding transcripts

The median 5′-UTR length is 52 nt, which is similar to that of *Synechocystis* sp. PCC 6803 [15], but longer than those of *Prochlorococcus* MED4 and MIT9313 (27 and 29 nt, respectively) [25]. Ninety-four gTSSs and thirty-three iTSSs were found to have a distance of less than 10 nt to the start codon (Additional file 1: Table S2). These TSSs were used to test the possibility of inaccurately annotated open reading frames (ORFs) or leaderless transcripts. A BlastP comparison showed that 21 ORFs had more than 45% homology with a shorter N-terminus; these ORFs were re-annotated. Another 19 gTSSs were considered to give rise to leaderless transcripts.

#### Differential transcription and stress response mechanisms

**Fig. 2:**
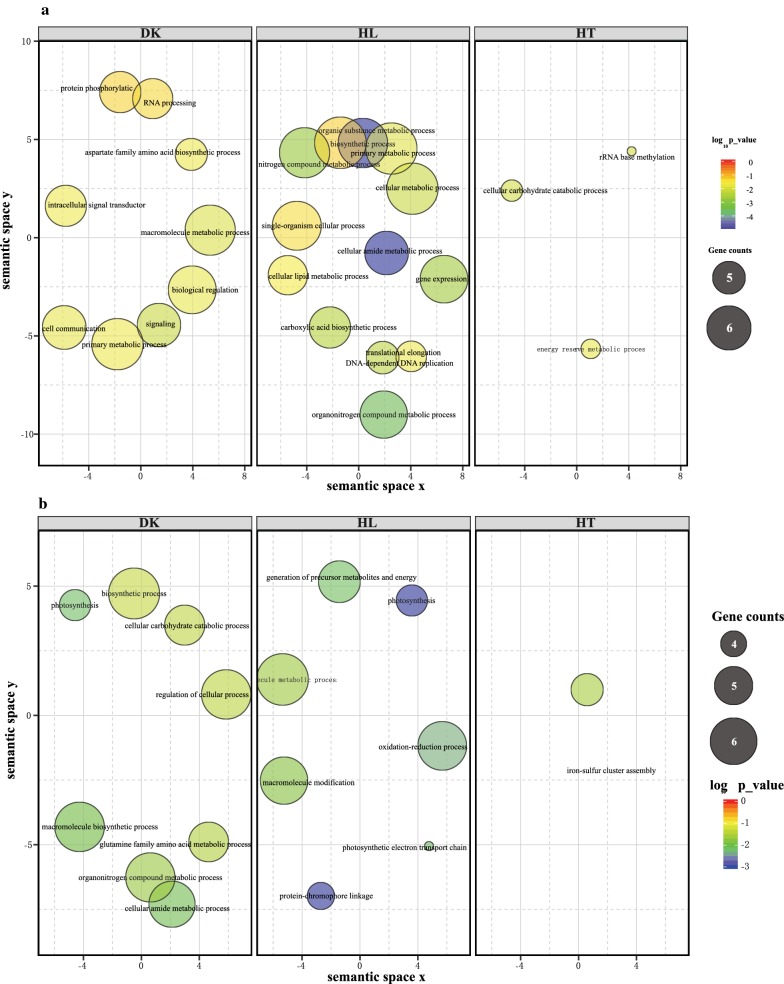
Overrepresented GO terms of up-regulated (**a**) and down-regulated genes (**b**) under the tested conditions. DK, darkness; HL, high light; HT, high temperature. The size of an enriched GO term represents the number of differentially expressed genes (Log_2_FC ≥ 1 and adjusted *p* value ≤ 1e−5) belonging to this GO term. Only GO terms with a *p* value ≤ 0.05 were used for semantic clustering using REVIGO [78]; redundant GO terms with a dispensability ratio ≥ 0.3 are not shown here but are available in Additional file 1: Table S3

The aggregated raw read counts of all TSSs (except for those of rRNAs and tRNAs) were normalized, and differential expression analysis was performed using DESeq2 [26] to investigate condition-dependent transcriptome remodeling. Transcripts with an absolute log_2_-fold change (Log_2_FC) ≥ 1 and an adjusted *p* value (padj) ≤ 0.01 were defined as differentially expressed transcripts (DET). The Log_2_FC and padj of each transcript under different conditions are summarized in Additional file 1: Table S1. Gene ontology (GO) and KEGG pathway enrichment analyses were conducted for differentially expressed protein-coding genes (DEGs). The results are shown in Fig. 2, Additional file 1: Tables S3 and S4.

#### Darkness induces dramatic transcriptome remodeling

The strongest transcriptomic differences were observed when the control was compared to the high-light and dark conditions, which accounted for the majority of variance shown in principal component analysis (PCA) analysis (Additional file 2: Figure S1). Altogether, 66.1% of the total set of TSSs were differentially transcribed under darkness (Additional file 1: Table S1 and Additional file 2: Figure S2). As shown in Additional file 1: Table S5, all of the top 10 up-regulated and 9 of the top 10 down-regulated transcripts showed their most dramatic transcript changes under darkness. The high proportions of abundant and differentially transcribed TSSs and the divergent transcriptome profiles between high-light and dark conditions indicate the occurrence of extensive transcriptional regulation during the light–dark transition of photoautotrophic *S. elongatus* UTEX 2973.

GO enrichment analysis showed that the genes up-regulated under darkness were mainly enriched in biological processes such as signal transduction, biological regulation, protein phosphorylation, and primary metabolic and macromolecule metabolic processes (Additional file 1: Table S3). In addition, KEGG enrichment analysis suggested that genes encoding the enzymes of glycerolipid (syu00561), pyruvate (syu00620) and glycogen (syu00500) metabolism pathways (Additional file 1: Table S4) were up-regulated in darkness. In contrast, the down-regulated genes were enriched in biological processes such as photosynthesis, translation and amino acid metabolism (Additional file 1: Table S3), which is consistent with the enriched KEGG pathway results (Additional file 1: Table S4).

**Fig. 3:**
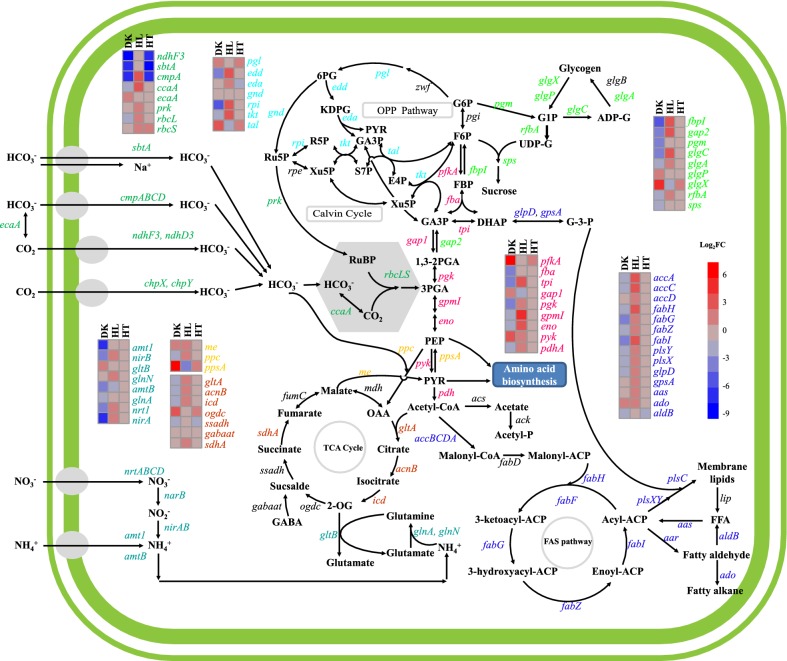
Effects of the applied conditions on the expression of central metabolism genes. The log_2-_fold change (Log_2_FC) is shown for each gene under dark, high-light and high-temperature conditions in this order on heat maps. The association of distinct genes to different pathways is color coded as follows. Carbon assimilation: dark green; OPP pathway: light green; gluconeogenesis, sucrose, and glycogen biosynthesis pathways: green; glycolysis: pink; anaplerotic reactions: gold; TCA cycle: red; nitrogen assimilation: dark cyan; and fatty acid, fatty alkanes, and membrane lipid biosynthesis pathways: blue. Abbreviations are listed in Additional file 1

Specific examples illustrate the physiological relevance of the detected changes in more detail. Two neighboring histidine kinase and regulator genes, M744_RS11880 and M744_RS11875, were highly induced under darkness (Additional file 1: Table S1), which is consistent with the regulation of their homologs in *S. elongatus* PCC 7942, Synpcc7942_0855 and Synpcc7942_0856 (PilH/Rre7) [27, 28]. Both proteins also possess a CheY-like domain. The co-transcription of these genes with the downstream chemotaxis protein CheW encoding gene (Supplementary dataset 1) suggests a connection between darkness acclimation and the regulation of motility. The carbon assimilation genes *ndhF3*, *sbtA*, *cmpA, ccaA* and *rbcL* and gluconeogenesis genes *pgk*, *fbpI* and *gap2* were repressed under darkness (Fig. 3), whereas the gene M744_RS01695 encoding the Calvin cycle inhibitor CP12 [29] was highly induced (Log_2_FC: 6.90) under the same condition (Table 1 and Additional file 1: Table S1). Meanwhile, most genes involved in photosynthesis and phycobilisomes were significantly down-regulated (Additional file 1: Table S6 and Additional file 2: Figure S3b ). These results suggest that photosynthetic carbon fixation is turned down under darkness (Fig. 3).

In addition, the *glgC* and *glgA* genes, encoding enzymes in glycogen biosynthesis, were also down-regulated under darkness, whereas the two genes encoding glycogen debranching enzyme (GlgX, Log_2_FC: 4.41) and glycogen phosphorylase (GlgP, Log_2_FC: 1.58) were significantly up-regulated (Fig. 3 and Additional file 1: Table S7). Thus, glycogen degradation was rapidly initiated, which probably fuels cellular metabolism in the absence of light energy. Regarding glycolysis-related genes, *pfkA* from the Embden–Meyerhof–Parnas (EMP) pathway was dramatically up-regulated (Log_2_FC: 6.5) under darkness. In addition, four glyceraldehyde-3-phosphate (GA3P) metabolism-related genes, including *eda* from the Entner–Doudoroff pathway (EDP) [30], *pgl a*nd *tal* from the oxidative pentose phosphate pathway (OPP), and *gap1* from the EMP pathway, were also up-regulated (Fig. 3 and Additional file 1: Table S7). These results suggest that glucose derived from glycogen was further oxidized via the EMP, EDP or OPP pathways in *S. elongatus* UTEX 2973. Furthermore, pyruvate metabolism-related genes, including *pyk*, *ppsA* and *me*, were significantly up-regulated (Log_2_FC: 2.6, 7.2 and 1.3, respectively) under darkness (Fig. 3 and Additional file 1: Table S7), indicating a need to replenish the pyruvate pool as a central intermediate in primary metabolism.

Transcripts of the big gene cluster encoding nitrite reductase and nitrate transporter (*nirA* and *nrtABCD*), originating from a single gTSS at position 1,999,635, were also dramatically repressed (Log_2_FC: − 6.64) under the darkness condition (Fig. 3 and Additional file 1: Table S7). Transcriptional down-regulation was also observed for the ammonia transporter genes *amt1* and *amtB* and for the glutamine synthetase (GS, EC 6.3.1.2) gene (*glnA*; Log_2_FC: − 1.95), indicating the inhibition of nitrogen assimilation under darkness (Fig. 3). In contrast, *gifA* encoding glutamine synthetase inactivating factor IF7 (M744_RS11650) was greatly up-regulated (Log_2_FC: 6.41) under darkness (Fig. 3 and Additional file 1: Table S7), which is consistent with microarray data reported for *S. elongatus* PCC 7942 [27]. The observed changes illustrate the need to turn down nitrogen assimilation under darkness.

It was unexpected that M744_RS11705 (*gltB*), encoding glutamate synthase subunit alpha (Ferredoxin-GOGAT, Fd-GOGAT), was slightly up-regulated (Log_2_FC: 0.8) and maintained as the 10th most abundant transcript in the darkness transcriptome (Table 1 and Fig. 3) because the same gene in *S. elongatus* PCC 7942 was significantly down-regulated under darkness [27]. Based on the redox-sensitive characteristic of Fd-GOGAT [31], it is assumed that Fd-GOGAT becomes inactivated under darkness. Comparative genomic analysis with PCC 7942 showed that there is a C3053T point mutation within the ORF of *gltB* in the genome of *S. elongatus* UTEX 2973, resulting in an S1018L substitution [4]. Our primary transcriptomic data showed that the position of this SNP is 231 and 295 nt away from an aTSS that was inversely regulated with *gltB* mRNA (Log_2_FC: − 2.05) and an iTSS, respectively. However, it is not clear whether the point mutation affects the transcription or stability of the *gltB* transcript.

As the third most abundant transcript in the dark transcriptome, *hspA*, which encodes a molecular chaperone, was up-regulated under darkness in both *S. elongatus* UTEX 2973 (Log_2_FC: 6.91; Table 1 and Additional file 1: Table S1) and PCC 7942 [27]. As a known cold-shock responding gene in *Anabaena* and *Synechocystis* [32–35], *rbp1* (M744_RS12780), encoding an RNA-binding protein, was significantly induced under darkness (Log_2_FC: 4.24) in *S. elongatus* UTEX 2973. Two other members of the RNA-binding protein family, *rbp2* (M744_RS06155) and *rbp3* (M744_RS12200), were significantly down-regulated (Log_2_FC < − 2) under darkness. This inverse regulation among members of the *rbp* family suggests that substantial shifts were occurring at a post-transcriptional level. Unfortunately, exact information on the function of these proteins is lacking.

#### The high-light responding transcripts

**Fig. 4:**
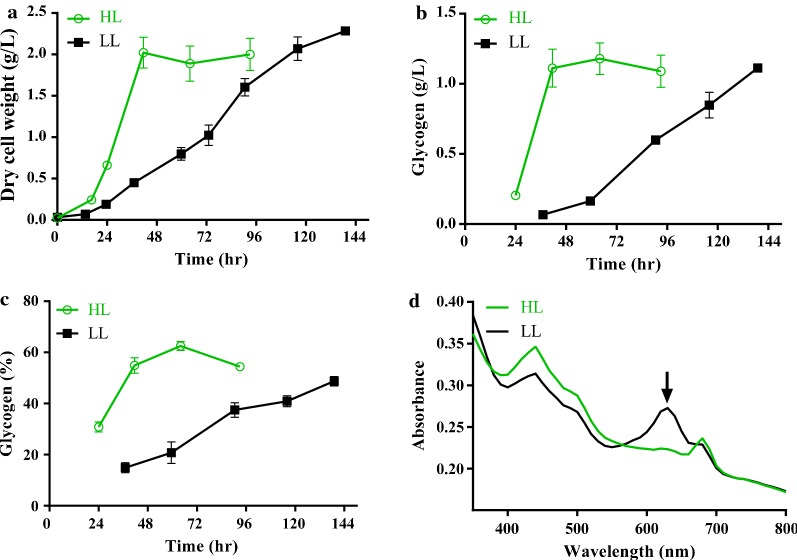
Biomass and glycogen accumulation of *S. elongatus* UTEX 2973 under low or high illumination. *S. elongatus* UTEX 2973 was grown in BG11 media at 41 °C with low-light (LL, 50 µmol photons, indicated by black lines and filled squares) or high-light conditions (HL, 500 µmol photons, indicated by blue lines and open circles). Both dry cell biomass production (**a**) and glycogen titers (**b**) of cultures were determined. Intracellular glycogen content (**c**) is shown as the percentages of dry cell weight. **d** Absorption spectra of cultures grown for 24 h; the phycobilisome absorption peak (625 nm) is indicated by the arrow. Error bars represent s.d. (*n* = 3)

*Synechococcus elongatus* UTEX 2973 grows faster at 38 °C with an illumination of 500 µmol photons/m^2^/s than with an illumination of 100 µmol photons/m^2^/s [4]. We observed a biomass accumulation rate of as much as 77.7 mg/L/h under high light (41 °C, 500 µmol photons/m^2^/s), which is 4.2-fold that of the accumulation rate under low light (41 °C, 50 µmol photons/m^2^/s; Fig. 4a) and is similar to previously reported values. Moreover, glycogen was accumulated at 54.9% of dry cell weight (DCW) with a rate of 51.9 mg/L/h under high light, which is 4.3-fold the rate in low-light conditions (Fig. 4b, c). Light absorption by phycobiliproteins was drastically reduced in cultures grown under high light for 24 h compared to growth at normal light (Fig. 4d). These findings show that high light significantly decreased phycobilisome content while enhancing both biomass and glycogen accumulation in *S. elongatus* UTEX 2973.

Under high light, 44.9% of all TSSs were differentially transcribed compared to the control conditions. The up-regulated protein-coding genes were mainly enriched in biological processes such as translation, amide, peptide biosynthesis and carboxylic acid metabolic processes (Additional file 1: Table S3), including pathways for the biosynthesis of amino acids or fatty acids, aminoacyl-tRNA, ribosomes and glycolysis/gluconeogenesis (Additional file 1: Table S4). The high light-repressed genes were enriched in biological processes such as protein-chromophore linkage, photosynthesis and oxidation–reduction processes, antenna proteins and photosynthesis.

Our dRNA-Seq results provide insights into the transcriptional changes between gene copies with identical coding sequences but divergent 5′-UTRs, promoters and regulation at high resolution. This finding is illustrated by the two identical copies of *cpcBA* genes (M744_RS10920-M744_RS10915; M744_RS10895-M744_RS10890). These four genes are part of a region in which nine phycobilisome-associated genes (M744_RS10880 to M744_RS10920) are clustered within ~ 6.5 kb, spanning the region from the gTSS at position 2,197,791 to the gTSS at position 2,204,398 (Additional file 2: Figure S3a). This region contains 20 TSSs, eight of which are aTSSs and four of which are nTSSs. We found that the transcriptional level of M744_RS10920-M744_RS10915 (*cpcB2*-*cpcA2*) was significantly higher than that of M744_RS10895-M744_RS10890 (*cpcB1*-*cpcA1*) under control, high-light and high-temperature conditions. The abundance of most transcripts that originated from gTSSs in this region decreased significantly under high light and darkness compared to the control (Additional file 1: Tables S1, S6 and Additional file 2: Figure S3), which is consistent with the reduction in phycobilisome content at high light and in darkness. This transcriptional regulation is likely supported by a post-transcriptional component mediated through the aTSSs at positions 2,200,086 and 2,203,841 within the two identical *cpcB* genes, which exhibited completely inverse regulation (Additional file 2: Figure S3a). Moreover, the nTSS at position 2,200,672 was dramatically induced (Log_2_FC: 4.95) by darkness (Additional file 1: Table S1), pointing to additional control through an sRNA.

Our results are consistent with reports on high light effects in *Synechocystis* sp. PCC 6803 [36]. Except for *psbAI*, two other *psbA* genes encoding form II of the photosystem II reaction center D1 protein were highly (at least fourfold) up-regulated by high light (Additional file 1: Table S6), which is in line with a previous report [37]. In addition, the *psbH* and *psb28* genes, encoding PSII reaction center proteins, were up-regulated threefold. Other genes encoding PSII proteins were not differentially transcribed in response to high light. In contrast, all genes encoding subunits of photosystem I (PSI) were down-regulated under the high-light condition (Additional file 1: Table S6 and Additional file 2: Figure S3b). Except for *petD*, all genes encoding subunits of the cytochrome b6/f complex were up-regulated. The *petF* and *petH* genes, encoding ferredoxin and ferredoxin-NADPH reductase, respectively, were up-regulated, whereas the *petE* and *petJ* genes, encoding electron transport carriers between cytochrome b6/f complex and photosystem I, were down-regulated. Genes encoding the beta, epsilon and c subunits of the F_o_F_1_ ATP synthase were up-regulated in response to the high-light stimulus (Additional file 1: Table S6). The results suggest that *S. elongatus* UTEX 2973 decreases light absorption by phycobilisomes, accelerates repair of the photodamaged PSII reaction center D1 protein, and increases NADPH and ATP synthesis under high light.

M744_RS13025 and M744_RS07280, encoding ribosomal proteins L10 and S21, were highly transcribed under high light, suggesting the acceleration of translation. As the third most abundant transcript in the high-light transcriptome, *sphX*, encoding a phosphate-binding protein, was up-regulated 4.0-fold. However, *somA*, encoding a major outer membrane porin [38], was highly transcribed and up-regulated (Log_2_FC: 2.6) by high light. The high abundance of these transcripts suggests fast assimilation of phosphate and other nutrients.

Several genes involved in carbon (*cmpA*, *ccaA*, *prk*, *rbcL* and *rbcS*) or nitrogen (*nirA*, *nirB* and *glnN*) assimilation, the OPP pathway (*rpi* and *tkt*), the EMP pathway (*tpi*, *gpmI*, *eno*, *pyk* and *pdhA*) and the TCA cycle (*gltA*, *acnB*, *icd* and *adhA*) were induced by high light. Additionally, two important gluconeogenesis genes, *gap2* and *fbpI*, were up-regulated. Meanwhile, glycogen biosynthesis genes, including *glgC* and *glgA*, were up-regulated at least threefold, whereas *glgP* and *glgX* were clearly inhibited (Fig. 3 and in Additional file 1: Table S7).

A group of genes involved in fatty acid biosynthesis (*accC*, *accD*, *fabH*, *fabA*, *fabG* and *fabI*), membrane lipid biosynthesis (*plsX* and *plsY*) and desaturation (*desC*) were up-regulated (Fig. 3 and Additional file 1: Table S7), indicating that membrane lipid biosynthesis was accelerated under high light. The up-regulation of *aas* and *ado* genes, encoding long chain fatty acid-ACP ligase and aldehyde-deformylating oxygenase, respectively, suggests that the fatty alkane biosynthesis pathway was also promoted. Considering the indispensable role of *aas* in high-light acclimation [39], fatty acid recycling and fatty alkane biosynthesis might be involved in high-light acclimation in cyanobacteria (Fig. 3 and Additional file 1: Table S7). Unlike what occurs in several other cyanobacteria [40], the *ado*-*aar* gene cluster was co-transcribed in *S. elongatus* UTEX 2973 (Supplementary Dataset S1 and S2).

Based on BlastN and GLASSgo sRNA homolog searching [41], the 131-nt sRNA with an nTSS at position 11,213 (CP006471) was identified as a homolog of the sRNA PsrR1, which was initially found in *Synechocystis* sp. PCC 6803 and governs essential steps of high-light acclimation [17] (Additional file 2: Fig. S4). An sRNA with the same length as PsrR1 was also found in the transcriptome of *S. elongatus* PCC 7942 (from 557,130 to 557,000, NC_007604; [42]). PsrR1 is highly induced by high-light illumination in both *S*. *elongatus* UTEX 2973 (Log_2_FC: 5.79; Table 1) and *Synechocystis* sp. PCC 6803 [21]. It was predicted or experimentally demonstrated that PsrR1 represses the translation of several genes encoding phycobilisome proteins, for example, *cpcA*, *cpcB*, *apcE*, and *apcF* [17]. In 2014, four works successively proved that minimizing the phycobilisome antenna by inhibition or reduction of *cpc* gene expression could dramatically improve the photosynthetic efficiency [43–45] of *Synechocystis* sp. PCC 6803, whereas *apcE* deletion promoted biomass and glycogen accumulation [46]. Therefore, the up-regulated sRNA PsrR1 plays a major role in resilience to high light.

#### High-temperature responding transcripts

Compared with the two conditions described above, only 10.9% of total TSSs were differentially transcribed under high-temperature conditions. The up-regulated genes were enriched in polysaccharide and glycogen catabolic processes, two-component system, and starch and sucrose metabolism pathways. The down-regulated genes were enriched in iron-sulfur and metallo-sulfur cluster assembly processes, photosynthesis and sulfur metabolism, and oxidative phosphorylation pathways.

Specifically, some genes involved in electron transport, such as *petF*, *petB* and *petC*, were down-regulated under high temperature. Additionally, some genes encoding the NAD(P)H-quinone oxidoreductase subunits (*ndhH*, *ndhA*, *ndhB* and *ndhC*) showed decreased transcript abundance, which might reflect decreased respiration under heat stress. In addition, sulfur transporter and sulfur transferase genes were found to be down-regulated, suggesting lowered sulfur assimilation under heat stress. Several inorganic carbon transporter genes (*ndhF3*, *sbtA*, and *cmpA*) were highly repressed by heat stress, whereas some nitrogen assimilation genes (*amt1*, *nirAB*, *amtB*, *glnA*, *glnN*, and *gltB*) showed similar levels to those under the normal conditions. In addition, *glgX*, encoding a glycogen debranching enzyme, was the only up-regulated glycogen metabolism gene, indicating the need to change the ratio of branched glycogen [47] in response to heat stress. Notably, a PSI gene (*psaA*) and two PSII genes (*psbI* and *psbE*) became the top 3 most abundant transcripts in the high-temperature transcriptome (Table 1 and Additional file 1: Table S1).

### A mis-annotated protein-encoding transcript with the highest abundance

gTSS-2349022 is linked to the most abundant RNA (except 16S rRNA) in the primary transcriptomes of *S. elongatus* UTEX 2973. Under darkness, the read count of this TSS accounted for as high as 12.5% of the total TSS counts (excluding 16S rRNA). Its transcript level was dramatically up-regulated (Log_2_FC: 7.30) after incubation under darkness for 2 h. Moreover, it was also strongly up-regulated under high-temperature conditions (Log_2_FC: 3.80) and down-regulated under high-light conditions (Log_2_FC: − 2.48). The TSS is located 785 bp upstream of the protein-encoding gene M744_RS11625. Because of the rather long distance, we speculated that the RNA covering this region is a separate transcript and might encode a protein. The following BLASTx and tBLASTn searches indeed identified a non-annotated open reading frame encoding 180 amino acids in this region that was identical to Synpcc7942_0905 (GenBank: ABB56935.1) of *S. elongatus* PCC 7942. The reported proteomes of *S. elongatus* PCC 7942 confirmed the translation of *Synpcc7942_0905* [48]. Moreover, there is a 100% identical homolog in *Synechococcus elongatus* PCC 6301. However, no homologs were found in other organisms besides these three closely related *Synechococcus* strains. We annotated this gene as protein-coding and assigned the locus name M744_RS11628 to conveniently cite this gene with a TSS at 2,349,022 and an ORF between M744-RS11625 and M744-RS11630.

Based on re-analysis of published transcriptomic data (Table S2 from [49] and Table S1 from [50]), the transcript level of *Synpcc7942_0905* was found to be most abundant among all protein-encoding genes of *S. elongatus* PCC 7942 under normal culture conditions (30 °C, 100 µmol photons/m^2^/s, CO_2_ aeration) and significantly changed by the tested treatments. These transcriptional analyses showed that *Synpcc7942_0905* is also a highly expressed and sensitive gene to environmental changes, similar to its homolog in *S. elongatus* UTEX 2973. No reported information about the function of this protein is available except that the first 30 amino acids likely constitute a signal peptide (probability of 1.000 with a cleavage site probability of 0.961) and that the gene is dispensable for *S. elongatus* PCC 7942 under laboratory conditions [51].

### Sye_sRNA1 is conserved in several cyanobacteria

In addition to protein-encoding genes, some sRNAs also quickly responded to the applied stimuli. Some previously identified sRNAs have high physiological relevance in cyanobacteria, for example, high light-induced PsrR1 [17], nitrogen-regulated NsiR4 [18] and iron starvation-induced IsaR1 [20].

Sye_sRNA1 is an sRNA starting from a TSS at position 1,559,116. Sye_sRNA1 was the most abundant sRNA (except 16S rRNA) in the transcriptomes of *S. elongatus* UTEX 2973 cultures grown under control and high-temperature conditions (Additional file 1: Table S1). The transcription of Sye_sRNA1 was somewhat inhibited by high light (Log_2_FC: − 1.42) and up-regulated in darkness (Log_2_FC: 1.35) (Table 1). We found 53 homologs of Sye_sRNA1 in the nr database using blastN analysis and the GLASSgo tool [41] (Additional file 2: Figure S5). Moreover, these homologs showed conserved secondary structures (Additional file 2: Figure S5b). Several of these homologs were experimentally confirmed, including those from *Anabaena* sp. PCC7120 [14], *S. elongatus* PCC 7942 [42], *Synechocystis* sp. PCC 6714 (TU2045) [16] and PCC 6803 (TU475) [15]. These homologs are also the most abundant transcripts (excluding 16S rRNA) and are repressed by high-light, high-temperature or dark stresses in both *Synechocystis* sp. PCC 6803 [15] and PCC 6714 [16]. Moreover, synteny analysis revealed that most of these Sye_sRNA1 homologs are located either downstream of a *psbD* or a *cobB* gene (Additional file 2: Figure S6). The sRNA homolog in *Synechocystis* sp. PCC 6803 even became incorporated into the coding region of *sll1501*, prolonging its coding region by 24 codons compared to the very close homolog in *Synechocystis* sp. PCC 6714.

**Fig. 5:**
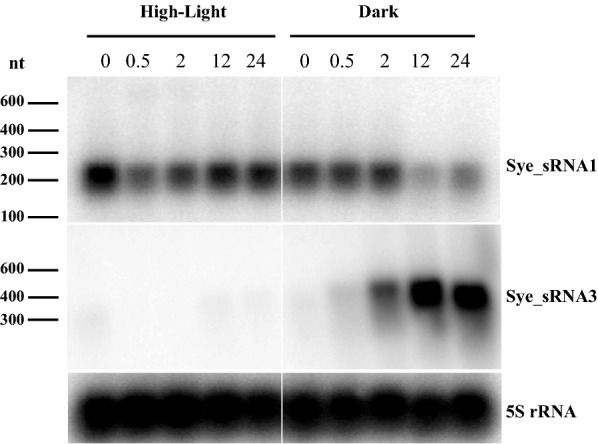
Northern blot analysis of Sye_sRNA1 and Sye_sRNA3 in *S. elongatus* UTEX 2973. Cells were initially grown at 33 °C with a light intensity of 150 µmol photons m^−2^ s^−1^ (CT) in the presence of 3% CO_2_ and were shifted to high-light conditions (HL, 700 µmol photons/m^2^/s) and dark conditions (DK, 0 µmol photons/m^2^/s). Total RNA was extracted at the given time points, blotted onto nylon membranes and hybridized with ^32^P labeled, specific RNA probes against Sye_sRNA1, Sye_sRNA3 and 5S rRNA

As shown in Fig. 5, the main transcript of Sye_sRNA1 is ~ 200 nt in length. Sye_sRNA1 was abundant under normal culture condition. After shifting to high light, the Sye_sRNA1 level transiently decreased within the first 2 h and then recovered to the initial level at 12 and 24 h. The high abundance of Sye_sRNA1 under normal condition and its obvious down-regulation in the early stage (0.5 h) of high-light acclimation are in accordance with the dRNA-Seq data (Table 1). After 12 h in darkness, Sye_sRNA1 expression was down-regulated.

### *Sye_sRNA3* is highly induced by darkness

Sye_sRNA3 (starting from position 1,471,309) was the most abundant sRNA (excluding 16S rRNA) after 2 h of darkness. The abundance of Sye_sRNA3 was significantly increased under darkness (Log_2_FC: 4.6) and high temperature (Log_2_FC: 1.3), but it decreased under high light (Log_2_FC: − 5.2). We did not find any homologs of Sye_sRNA3. Northern blot analysis of Sye_sRNA3 showed a transcript of almost 500 nt that appeared at 2 h in the dark, dramatically increased in signal intensity after 12 h (Fig. 5), and rapidly disappeared in the light (Additional file 2: Figure S7).

### asRNAs and their potential targets

In our primary transcriptomes, 1380 aTSSs were identified, accounting for 28.59% of all TSSs (Fig. 1a). We compared and statistically analyzed the transcriptional changes of asRNAs and their corresponding mRNAs. We found 77, 21 and 3 asRNAs that were inversely regulated with their potential targets under dark, high-light and high-temperature conditions, respectively (Additional file 1: Table S8). The functions of the target genes were widely distributed, including gene regulation (*sigF2* and *sigB*), energy metabolism (*ndhA*, *psbA2*, *atp1* and *atpH*), replication, translation and cell division (*gyrA*, *dnaA*, *rpl17*, *rps1* and *ftsZ*), and glycerol metabolism (*gpsA* and *gldA*). Our previous reports also experimentally proved the roles of asRNAs in the regulation of cyanobacterial gene expression [52–54]. Based on this inverse regulation and the fact that several cyanobacterial asRNAs play regulatory roles, it is very likely that some of the identified cyanobacterial asRNAs are functionally relevant and not simply the result of transcriptional noise [55].

## Discussion

In this study, we employed dRNA-Seq to characterize and compare the primary transcriptomes of *S. elongatus* UTEX 2973 under different culture conditions. Compared with classical RNA-Seq approaches, an additional TEX digestion step is performed in dRNA-Seq library construction [13], which enables precise and genome-wide determination of TSSs at single-nucleotide resolution. This method is superior for the identification of sRNAs, which is exemplified by our finding of 229 nTSSs that give rise to such potential post-transcriptional regulators. The vast majority of bacterial sRNAs are transcribed from their own genes, i.e., free-standing genes within the intergenic space between two protein-coding genes. However, some sRNAs were found to originate from the 3′-regions or 3′-UTRs of mRNA [56]. Our synteny analysis suggests that the homolog of Sye_sRNA1 in at least one other species became fused to the 3′ end of the *cobB* reading frame. Hence, this finding illustrates the possible trajectory for the evolutionary origin of such 3′-UTR-derived sRNAs by fusion of the sRNA with its own promoter at the end of a reading frame.

However, primary transcriptome information is most relevant for all attempts at genetic manipulation and is especially meaningful for metabolic engineering aimed at biotechnological applications. First, some ORFs were found to host the TSSs of downstream genes or intragenic transcripts or TSSs that gave rise to antisense RNAs. These arrangements can be highly relevant. For instance, in *Synechocystis* sp. PCC 6803, a sl*r1588* deletion mutant with an intragenic TSS affected the transcription of downstream gene *ggpP*, leading to an unexpected salt-sensitive phenotype [57]. Hence, detailed TSS information is crucial for the accurate evaluation of gene functions by gene deletion or over-expression. Second, many genes are associated with more than one TSS. The alternative use of different TSSs provides insights into underlying gene regulation under different environmental conditions as impressively illustrated by our analysis of the phycocyanin gene cluster. Third, transcriptomic information can be integrated into constraint-based metabolic models to improve metabolic flux predictions using multiple established approaches [58].

As photoautotrophic microorganisms, cyanobacteria depend on light energy to support their growth. In natural environments, they go through daily light–dark transitions and have evolved efficient mechanisms to adjust gene expression according to these changes [59]. Under darkness, changes in redox (quinone pool) and energy status (ATP/ADP) would be sensed by the regulators KaiA, CikA and RpaA, where RpaA controls the transcription of downstream genes [27, 59, 60], such as *psbI*, *psbE*, *gifA* and *hspA*, in order to regulate carbon and nitrogen metabolism. However, not every dark-induced or repressed gene is regulated by these circadian rhythm regulators, such as the *gltB* gene, which encodes Fd-GOGAT [27]. In the present work, the *gltB* gene was unexpectedly found to be highly transcribed under darkness in *S. elongatus* UTEX 2973. The *rbp1* gene was proven to be repressed in the dark condition under the regulation of KaiABC in *S. elongatus* PCC 7942 [27], but it was greatly up-regulated in *S. elongatus* UTEX 2973 (Table 1). The unusual transcription of these genes might be due to some unknown regulatory mechanisms in *S. elongatus* UTEX 2973. In addition, a protein-encoding gene (M744_RS11628) and two abundant sRNAs (Sye_sRNA1 and 3) were all up-regulated under darkness. As reported for other cyanobacteria [61, 62], glycogen in *S. elongatus* UTEX 2973 would be degraded and oxidized to compensate for intracellular energy and reductant demands under darkness through the OPP, EMP or EDP pathways.

Cyanobacterial photosystem complexes, especially PSII, become inevitably damaged by light [63, 64] by performing photosynthesis [65, 66]. Under high-light stress, cyanobacteria lower the excess light energy transferred to the PSII center by non-photochemical quenching (NPQ), decreasing the biosynthesis of phycobilisomes and accelerating the turnover of D1 protein [65, 66]. Artificial truncation of phycobilisome antenna sizes in *Synechocystis* sp. PCC 6803 decreased the light energy transferred to the PSII center and increased light absorption efficiency [44]. The latter changes the energy distribution between PSII and PSI, further re-modulates core cellular mechanisms [67], and finally increases photosynthetic carbon fixation efficiency and biomass accumulation [44, 46]. Aside from the demonstrated PsrR1-mediated post-transcriptional regulation [17], *cpc* operons could also be regulated by the NblS-RpaB two-component system [68]. The mechanism leading to phycobilisome regulation needs further investigation as the phycobilisome represents an important photosynthesis antenna complex. Information on multiple gTSSs and asRNAs of the *cpc* gene cluster (Additional file 1: Tables S6, S8 and Additional file 2: Figure S4a) provides a solid basis for further investigation in *S. elongatus* UTEX 2973.

## Conclusions

The dRNA-seq technique was employed to comparatively analyze the primary transcriptomes of *S. elongatus* UTEX 2973 under normal, high-light, high-temperature and dark conditions. In total, we accurately identified 4808 TSSs from these transcriptomes and evaluated transcriptional responses of all transcripts including some sRNAs under different stress conditions. Also, we experimentally verified the transcription of two abundant sRNAs and found that one of them (Sye_sRNA1) is widely conserved in cyanobacteria, whereas another one (Sye_sRNA3) is only transcribed under dark. Thus, this study provided detailed information about the genome-wide mapped TSSs, sRNAs and promoter activities of *S. elongatus* UTEX 2973, which are valuable for future research on metabolic engineering and into stress acclimation of this strain.

## Methods

### Strains and culture conditions of *S. elongatus* UTEX 2973

*Synechococcus elongatus* UTEX 2973 was obtained from the Culture Collection of Algae at The University of Texas at Austin (UTEX). *S. elongatus* UTEX 2973 was inoculated at an initial OD_730_ of 0.05 in BG-11 medium and subjected to standard (control) culture conditions (33 °C, 50 μmol photons/m^2^/s constant illumination, 3% CO_2_ aeration). To test transcriptomic responses to different stresses, exponential phase cultures (OD_730_ ≈ 0.5) were transferred to three different conditions: (i) high light, 1000 μmol photons/m^2^/s for 30 min; (ii) high temperature, 45 °C for 30 min; and (iii) darkness, no illumination for 2 h. For transcriptomic sequencing, 30 mL of cultures with or without treatments was collected, and total RNA was extracted, treated by DNase I, and sent to vertis Biotechnologie AG (Freising, Germany) for primary and random cDNA library constructions and next-generation sequencing. For Northern blot analysis, exponential phase cultures were shifted to high light (700 μmol photons/m^2^/s) or darkness, and 10 mL aliquots were collected at different time points for the isolation of total RNA.

For the determination of biomass and glycogen accumulation rates, *S. elongatus* UTEX 2973 was initially inoculated at OD_730_ ≈ 0.05 and grown at 41 °C in the presence of 3% CO_2_ aeration. High-light cultures were incubated at a light intensity of 1000 µmol photons/m^2^/s, whereas controls were incubated at 50 µmol photons/m^2^/s. Cell growth was monitored by measuring OD_730_ and converted to dry cell biomass by pre-established calibration [5]. Glycogen content was determined as previously described [5].

### RNA extraction, cDNA synthesis and sequencing

Cells sampled from four differential conditions were filtered on hydrophilic polyethersulfone filters (Pall, 0.8 μm, Port Washington, USA). The filters with cells were rapidly immersed in liquid nitrogen and ground to a fine powder in liquid nitrogen. Total RNA was isolated by TRIzol Reagent (Life Technologies, USA) according to the manufacturer’s instructions and treated with RNase-free DNase I (Takara, Japan) to eliminate contaminating chromosomal DNA.

Three different6 types of cDNA libraries were constructed: primary libraries, minus libraries and a reference library.

Primary libraries: For dRNA-Seq analysis, primary cDNA libraries were prepared by vertis Biotechnologie AG, Germany, as previously described [69] with the following modifications. Total RNA (10–20 µg) from each of the two replicate cultures was fragmented by ultrasound (4 pulses of 30 s at 4 °C). The resulting fragments were treated with T4 polynucleotide kinase (NEB, USA) and terminator-5′P-dependent-exonuclease (TEX) to enrich 5′PPP-containing primary transcripts and remove processed and partly degraded RNAs. Primary cDNA libraries were constructed following treatment by RNA 5′ pyrophosphatase (+5′PP, epicenter) to generate 5′ monophosphates and ligation of a 5′-RNA-adapter by cDNA synthesis using oligo-dT primers containing a 3′-adapter and 14–16 cycles of PCR amplification.

Minus libraries: Minus cDNA libraries were constructed for each RNA sample by following the same protocol but omitting the +5′PP digestion. These libraries were used as negative controls for each corresponding primary cDNA library.

Reference library: Aliquots of all 16 RNA samples were pooled and depleted of ribosomal RNA (rRNA) using the Ribo-Zero rRNA Removal Kit (Illumina, USA) to estimate the coverage of each transcript. The rRNA-depleted RNA samples were fragmented using ultrasound (4 pulses of 30 s each at 4 °C). Then, an oligonucleotide adapter was ligated to the 3′ RNA ends. First-strand cDNA synthesis was performed using an M-MLV reverse transcriptase with a primer complementary to the 3′-adapter. First-strand cDNA was purified, ligated to the 5′ Illumina TruSeq sequencing adapter, and amplified by 11 cycles of PCR.

All cDNA pools were sequenced on an Illumina NextSeq500 system using a 75-bp read length. The raw reads were deposited in the National Center for Biotechnology Information (NCBI) BioProject database (Accession Number: PRJNA420395).

### Reads mapping and TSS calling

The detailed bioinformatics workflow was described by Hou et al. [23, 70]. Briefly, the adapters of raw reads were removed using Cutadapt v1.0 [71], and then fastq_quality_trimmer (from FASTX-Toolkit v0.0.13, available at http://hannonlab.cshl.edu/fastx_toolkit/) was applied to trim and remove low quality reads; the resultant clean reads were converted into FASTA format and clustered into unique representative clusters with 100% identity. FastQC v0.10.1 [72] was used to perform a quality check of both raw and clean reads. SortMeRNA v1.9 [73] was applied to remove rRNAs. The remaining non-rRNA reads were aligned to the UTEX 2973 reference genome (CP006471, CP006472 and CP006473) using segemehl v0.2.0 [74] at 95% identity.

We employed a replicate-assisted background subtraction algorithm for TSS identification, which accounted for both the replicates and background information. The detailed algorithm was described by Hou et al. [70], and an example implementation of this algorithm can be found at https://github.com/housw/GRPutils. We followed the standard TSS classification practice [22] that defines gTSSs as TSSs located within 200 nt upstream of annotated protein-coding genes or initiating reads overlapping these genes. An iTSS or aTSS was assigned when a TSS located within an annotated gene or antisense to it in a range of 50 nt up- and downstream, respectively. When a TSS was found in front of a non-coding RNA, such as an sRNA, rRNA and tRNA, or in an intergenic region but was not claimed by the other TSS types, an nTSS was assigned. The whole pipeline, required software dependences and input data have been integrated into a docker image for reproducible research, which can be found at https://hub.docker.com/r/shengwei/utex2973_primary_transcriptome/.

### Differential expression and enrichment analyses

The clean non-rRNA reads from all dRNA-Seq libraries were summarized into identified TSS positions to detect differentially expressed TSSs. Only TSSs with a minimum of raw reads ≥ 300 at one of the libraries were kept, and TSSs initializing rRNAs and tRNAs were excluded from the following analysis. The count table was normalized, and differentially expressed TSSs (DET) were identified using DESeq2 [26] with an absolute log_2_-fold change (Log_2_FC) ≥ 1 and an adjusted *p* value ≤ 0.01.

For GO enrichment analysis, the GO terms for each protein-coding gene of UTEX 2973 were obtained using Blast2GO v4.1.7 [75] with an input GO mapping result generated by InterProScan v5.26-65.0 [76] and input BlastP result against NCBI non-redundant protein database (nr, downloaded in Oct. 2017). The significances of overrepresented GO terms were tested using the GOstats package v2.42.0 [77] under a hypergeometric distribution. The resulting p values were corrected with the qvalue v2.8.0 package (available at https://github.com/StoryLab/qvalue) for multiple-testing adjustment. Please note that the *q* values might be too stringent in this case since the GO terms are not independent of each other; therefore, *p* values were used in the following analysis. The enriched GO terms were semantically clustered using the REVIGO [78] online server with the default parameters and visualized with the ggplot2 v2.2.1 package [79] for GO terms with a dispensability ratio ≤ 0.3.

For KEGG pathway enrichment analysis, only gTSSs with a Log_2_FC ≥ 1 and an adjusted *p* value ≤ 1e−5 were used. KEGG pathway enrichment was done using clusterProfiler v3.4.4 [80], and enriched pathways were visualized using pathview v1.16.7 [81] with the DESeq2-generated Log_2_FC table. When multiple gTSSs mapped to the same gene, only the one with the lowest adjusted *p* value was used for GO and KEGG enrichment analyses, and only the one with maximum expression across all libraries was used for pathway visualization.

### Leaderless transcripts and mis-annotated ORFs

gTSSs with a distance of less than 10 nt from the start codon and iTSS were selected for further analysis. Their corresponding ORFs were used as queries for blast analyses against the NCBI nr protein database. The *e* value cutoff was set to 1E−5. If more than 45% of the homologs showed a matching start position, the query transcript (gTSS) was considered to give rise to a leaderless transcript. If more than 45% of homologs showed a start position leading to shorter protein, then re-annotation of the start codon of the query ORF was proposed.

### Northern blot analysis

Total RNA was isolated by the PGTX method [82]. Approximately, 3 μg of total RNA was separated on 1.5% agarose gels with 16% formaldehyde, transferred to Hybond N^+^ nylon membranes (Amersham) by capillary blotting, cross-linked by UV-illumination (Stratalinker 2400, Stratagene), and hybridized by [γ-^32^P] ATP-labeled RNA probes as previously described [15]. RNA probes were produced from PCR-generated templates using in vitro transcription (MaxiScript kit, Ambion). All DNA oligonucleotides used for PCR and hybridization experiments were ordered from Sigma-Aldrich (Hamburg, Germany) (Additional file 1: Table S9). Signals were visualized using a Typhoon FLA 7000 system (Amersham).

## Additional files

**Additional file 1: Table S1.** All information of each identified TSS. **Table S2.** Leaderless transcripts and mis-annotated genes. **Table S3.** GO terms enrichments. **Table S4.** KEGG pathway enrichments. **Table S5.** Top 10 up-regulated and top 10 down-regulated transcripts. **Table S6.** Transcript changes of genes associated with photosynthesis and phycobilisomes. **Table S7.** Transcript changes of genes associated with central metabolisms. **Table S8.** asRNAs and their potential target genes. **Table S9.** List of primers used for the amplification of probe templates.

**Additional file 1: Table S1.** **Figure S1.** Principal component analysis (PCA) of the primary transcriptomes of different conditions. **Figure S2.** Volcano and MA plots of differentially transcribed TSSs from the transcriptomes of *S. elongatus* UTEX 2973 grown under different conditions. **Figure S3.** TSS distributions in the two *cpcBA* gene clusters and effects of different stresses on the transcription of genes associated with photosynthesis and phycobilisomes. **Figure S4.** Multiple sequence alignment of PsrR1 homologs from selected cyanobacteria. **Figure S5.** Sequence and structure conservation of Sye_sRNA1. **Figure S6.** Synteny map for the region surrounding the Sye_sRNA1 of cyanobacteria. **Figure S7.** Accumulation of Sye_sRNA3 in *S. elongatus* UTEX 2973 grown under light–dark transition conditions.
