# Supplementary material for: The primary transcriptome of the fast-growing cyanobacterium Synechococcus elongatus UTEX 2973
Source: Biotechnol Biofuels. 2018 Aug 4;11:218. doi: 10.1186/s13068-018-1215-8 (PMC6091082; doi:10.1186/s13068-018-1215-8)
Supplement: Supplementary file 2 — Additional file 1: Table S1. Figure S1. Principal component analysis (PCA) of the primary transcriptomes of different conditions. Figure S2. Volcano and MA plots of differentially transcribed TSSs from the transcriptomes of S. elongatus UTEX 2973 grown under different conditions. Figure S3. TSS distributions in the two cpcBA gene clusters and effects of different stresses on the transcription of genes associated with photosynthesis and phycobilisomes. Figure S4. Multiple sequence alignment of PsrR1 homologs from selected cyanobacteria. Figure S5. Sequence and structure conservation of Sye_sRNA1. Figure S6. Synteny map for the region surrounding the Sye_sRNA1 of cyanobacteria. Figure S7. Accumulation of Sye_sRNA3 in S. elongatus UTEX 2973 grown under light–dark transition conditions. [file 13068_2018_1215_MOESM2_ESM.docx]

**Abbreviations**

**Genes:**

*aar*, long-chain acyl-[acyl-carrier-protein] reductase; *aas*, long-chain fatty acid--CoA ligase; *accA*, acetyl-CoA carboxylase subunit alpha; *accB*, acetyl-CoA carboxylase biotin carboxyl carrier protein; *accC*, acetyl-CoA carboxylase, biotin carboxylase subunit; *accD*, acetyl-CoA carboxylase carboxyl transferase subunit beta; *ack*, acetate kinase; *acnB*, aconitate hydratase 2; *acs*, acetyl-CoA synthetase; *ado*, aldehyde oxygenase (deformylating); *aldB*, aldehyde dehydrogenase family protein; *amt1*, ammonium transporter; *amtB*, ammonium transporter; *ccaA*, carbonic anhydrase; *ccmM*, carbon dioxide concentrating mechanism protein CcmM; *chlG*, chlorophyll synthase ChlG; *chpX*, carbon dioxide transporter; *chpY*, carbon dioxide transporter; *cmpA*, bicarbonate-binding protein CmpA; *cmpB*, bicarbonate ABC transporter permease; *cmpC*, bicarbonate transport ATP-binding protein CmpC; *cmpD*, bicarbonate transport ATP-binding protein CmpD; *ddh*, lactate dehydrogenase; *ecaA*, carbonic anhydrase; *eda*, KDPG aldolase; *edd*, phosphogluconate dehydratase; *eno*, enolase; *fabD*, malonyl CoA-ACP transacylase; *fabF*, 3-oxoacyl-ACP synthase; *fabG*, 3-ketoacyl-ACP reductase; *fabH*, 3-oxoacyl-[acyl-carrier-protein] synthase III; *fabI*, enoyl-ACP reductase; *fabZ*, 3-hydroxyacyl-ACP dehydratase; *fba*, fructose-1,6-bisphosphate aldolase; *fbpI1*, fructose 1,6-bisphosphatase; *fbpI2*, fructose 1,6-bisphosphatase; *fumC*, fumarate hydratase; *gap1*, glyceraldehyde-3-phosphate dehydrogenase 1; *gap2*, glyceraldehyde-3-phosphate dehydrogenase 2 ; *glgA*, glycogen synthase; *glgC*, glucose-1-phosphate adenylyltransferase; *glgP*, glycogen phosphorylase; *glgX*, glycogen debranching enzyme; *glnA*, glutamine synthetase; *glnN*, glutamine synthetase; *glpD*, glycerol-3-phosphate dehydrogenase; *gltA*, citrate synthase; *gltB*, ferredoxin-dependent glutamate synthase; *gnd*, 6-phosphogluconate dehydrogenase; *gpmI*, phosphoglycerate mutase; *gpsA*, glycerol-3-phosphate dehydrogenase; *icd*, isocitrate dehydrogenase; *me*, malate dehydrogenase; *narB*, nitrate reductase(ferredoxin); ndhD3, NAD(P)H-quinone oxidoreductase subunit D4; *ndhF3*, oxidoreductase; *nirA*, ferredoxin--nitrite reductase; *nirB*, nitrite reductase; *nrt1*, taurine ABC transporter substrate-binding protein; *nrt2*, nitrate ABC transporter permease; *nrtA*, nitrate transport ATP-binding protein NrtA; *nrtB*, nitrate transport ATP-binding protein NrtB; *nrtC*, nitrate transport ATP-binding protein NrtC; *nrtD*, nitrate transport ATP-binding protein NrtD; *pdhA*, pyruvate dehydrogenase (acetyl-transferring) E1 component subunit alpha; *pfkA*, 6-phosphofructokinase; *pgi*, glucose-6-phosphate isomerase; *pgk*, phosphoglycerate kinase; *pgl*, 6-phosphogluconolactonase; *pgm*, phosphoglucomutase; *plsC*, acyl-phosphate glycerol 3-phosphate acyltransferase; *plsX*, glycerol-3-phosphate acyltransferase; *plsY*, glycerol-3-phosphate acyltransferase; *ppc*, phosphoenolpyruvate carboxylase; *ppsA*, phosphoenolpyruvate synthase; *prk*, phosphoribulokinase; *pyk*, pyruvate kinase; *rbcL*, ribulose 1,5-bisphosphate carboxylase large subunit; *rbcS*, ribulose 1,5-bisphosphate carboxylase small subunit; *rfbA*, Glucose-1-phosphate thymidylyltransferase; *rpe*, ribulose-phosphate 3-epimerase; *rpi*, ribose-5-phosphate isomerase; *sbtA*, sodium-dependent bicarbonate transport family permease; *sdhA1*, succinate dehydrogenase / fumarate reductase, cytochrome b subunit; *sdhA2*, succinate dehydrogenase / fumarate reductase, iron-sulfur subunit; *sdhA3*, succinate dehydrogenase / fumarate reductase, flavoprotein subunit; *sps*, HAD family hydrolase; *tal*, transaldolase; *tkt*, transketolase; *tpi*, triosephosphate isomerase; *zwf*, glucose-6-phosphate dehydrogenase

**Compounds:**

3PGA, 3-phosphoglycerate; 6PG, 6-P-gluconate; DHAP, dihydroxyacetone phosphate; DMAPP, dimethylallyl pyrophosphate; F6P, fructose 6-phosphate; FNR, ferredoxin NADP+ reductase; G1P, glucose 1-phosphate; G6P, glucose 6-phosphate; GA3P, glyceraldehyde 3-phosphate; PEP, phosphoenolpyruvate; PYR, pyruvate; Ru5P, ribulose 5-phosphate; RuBP, ribulose 1, 5-bis-phosphate; Sucsalde, Succinyl semialdehyde


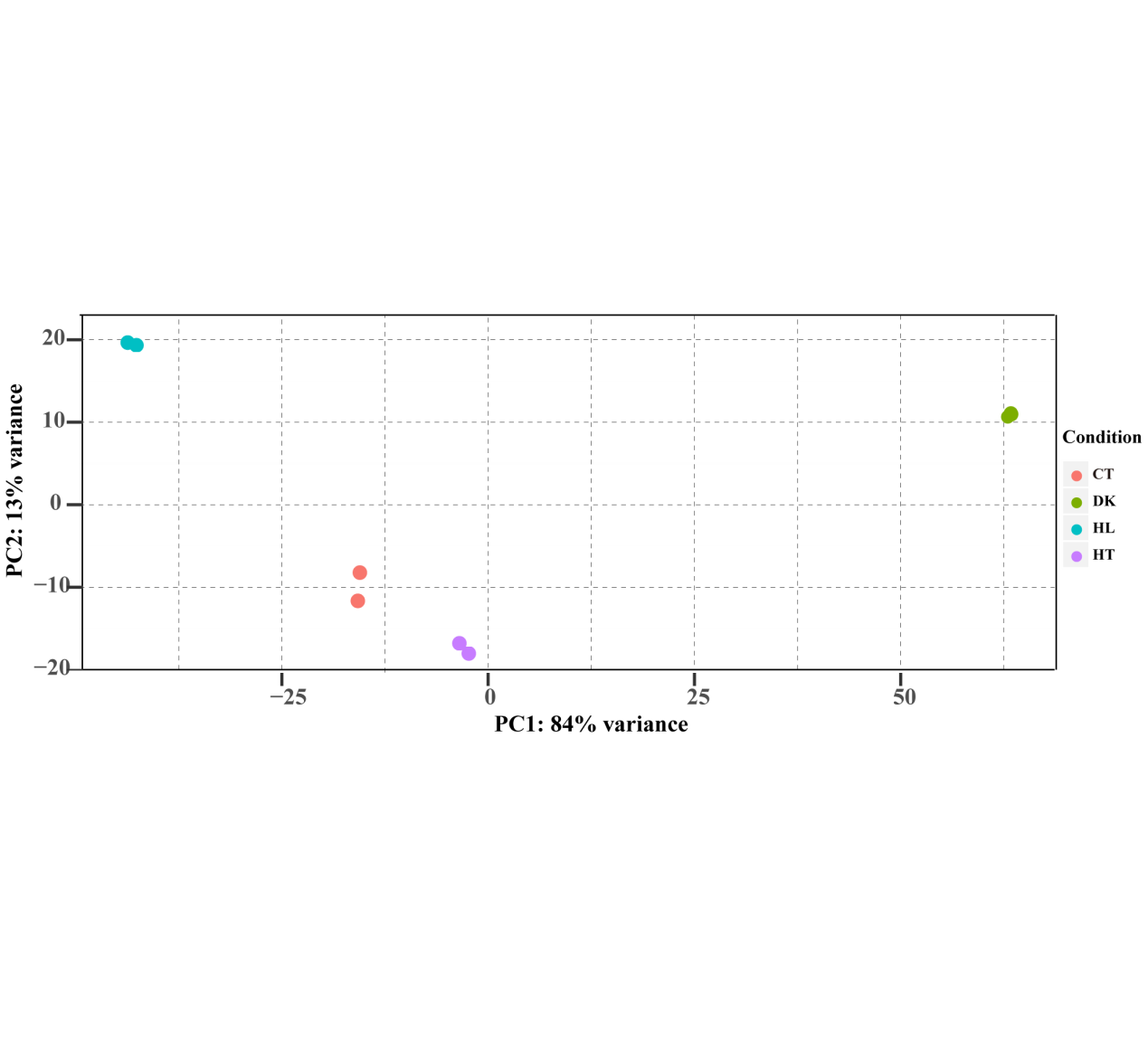


**Figure S1. Principal component analysis (PCA) of the primary transcriptomes of different conditions.** Only the top 500 TSSs with highest row variance were selected for this analysis using DESeq2 [1].

**Figure S2. Volcano and MA plots of differentially transcribed TSSs from the transcriptomes of *S. elongatus* UTEX 2973 grown under different conditions.** Panels a, b and c, represent the volcano plots of differentially transcribed TSSs under dark, high-light and high-temperature conditions, respectively. Panels d, e and f represent MA plots of differentially transcribed TSSs under dark, high-light and high-temperature conditions, respectively. The horizontal lines indicate the significance threshold (padj <= 0.01), whereas the vertical lines indicate the two-fold change threshold. Differentially transcribed TSSs with two-fold changes and padj no more than 0.01 are shown as black dots, whereas other TSSs are shown as red dots.

**Figure S3. TSS distributions in the two *cpcBA* gene clusters and effects of different stresses on the transcription of genes associated with photosynthesis and phycobilisomes.** a, TSS distributions over the *S. elongatus* UTEX 2973 genomic region (ranging from 2196976 to 2205203), which encodes two *cpc* gene clusters. Color-coded plots represent the normalized reads from primary transcriptome libraries of *S. elongatus* UTEX 2973 grown under 4 tested conditions. Coding DNA sequences (CDS) are shown in blue boxes, whereas TSSs are indicated as red arrows. Genes in the *cpc* gene clusters are shown as their gene symbols (*cpcF*, *E*, *A2*, *B2*, *D*, *C2*, *C1*, *A1* and *B1* represent M744_RS10880, 10885, 10890, 10895, 10900, 10905, 10910, 10915 and 10920, respectively). Each TSS is labeled by its TSS_ID in red font, and the complete information for each TSS is summarized in Table S1. b-c, Heat maps showing effects of stresses on the transcription of genes associated with photosynthesis and phycobilisomes. The genes were mapped to KEGG pathways, and the normalized Log_2_FC values compared to control conditions are shown as heatmaps using the Pathview [2] package. The transcriptional changes of each mapped gene under darkness (DK), high light (HL) and high temperature (HT) are shown as three neighboring squares.


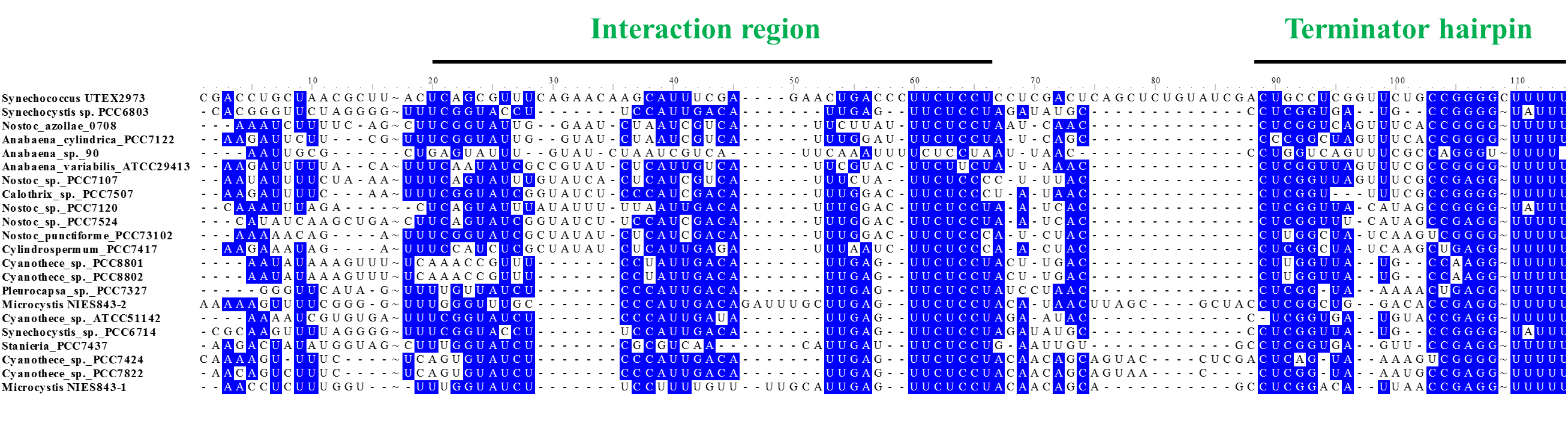


**Figure S4. Multiple sequence alignment of PsrR1 homologs from selected cyanobacteria**. Homologs (except for the *S. elongatus* UTEX 2973 sequence) were taken from a previous report [3].

**Figure S5. Sequence and structure conservation of Sye_sRNA1.** a, Multiple sequence alignment of Sye_sRNA1 homologs from selected cyanobacterial strains. b, Conserved secondary structure inferred from the sequence alignment. The structure was predicted using RNAalifold [4] from ViennaRNA v2.1.9 [5] and visualized using R2R v1.0.4 [6].

**Figure S6. Synteny map for the region surrounding the Sye_sRNA1 of cyanobacteria**. Each protein-encoding gene is represented by a rectangle. The *cob* genes encode cobyrinic acid a,c-diamide synthase.

**Figure S7. Accumulation of Sye_sRNA3 in *S. elongatus* UTEX 2973 grown under light-dark transition conditions.** Exponential phase cultures of *S. elongatus* UTEX 2973 were shifted to darkness for 24 h and then re-exposed to light. Samples were taken at different time points for Northern blot analysis.

**References**

1. Love MI, Huber W, Anders S. Moderated estimation of fold change and dispersion for RNA-seq data with DESeq2. Genome Biol 2014;15(12):550.

2. Luo W, Brouwer C. Pathview: an R/Bioconductor package for pathway-based data integration and visualization. Bioinformatics 2013;29(14):1830-1831.

3. Georg J, Dienst D, Schurgers N *et al*. The small regulatory RNA SyR1/PsrR1 controls photosynthetic functions in cyanobacteria. Plant Cell 2014;26(9):3661-3679.

4. Bernhart SH, Hofacker IL, Will S *et al*. RNAalifold: improved consensus structure prediction for RNA alignments. BMC Bioinformatics 2008;9(1):474.

5. Lorenz R, Bernhart SH, Höner zu Siederdissen C *et al*. ViennaRNA Package 2.0. Algorithms for Molecular Biology : AMB 2011;6:26-26.

6. Weinberg Z, Breaker RR. R2R - software to speed the depiction of aesthetic consensus RNA secondary structures. BMC Bioinformatics 2011;12:3-3.
